# Advanced screening methods for assessing motility and hatching in plant-parasitic nematodes

**DOI:** 10.1186/s13007-024-01233-z

**Published:** 2024-07-20

**Authors:** Alena Kadlecová, Romana Hendrychová, Tomáš Jirsa, Václav Čermák, Mengmeng Huang, Florian M.W. Grundler, A. Sylvia S. Schleker

**Affiliations:** 1 Department of Experimental Biology, Faculty of Science, Palacký University, Šlechtitelů 27, Olomouc, CZ-78371 Czech Republic; 2 Molecular Phytomedicine, University of Bonn, Karlrobert-Kreiten-Straße 13, 53115 Bonn, Germany; 3 Department of Plant Pests Diagnostics, Central Institute for Supervising and Testing in Agriculture, Šlechtitelů 773/23, Olomouc, CZ-77900 Czech Republic

**Keywords:** Plant-parasitic nematodes, *Heterodera schachtii*, *Ditylenchus destructor*, Protocol, Motility, Hatching, WMicrotracker ONE, Chitinase

## Abstract

**Background:**

Plant-parasitic nematodes are economically important pests responsible for substantial losses in agriculture. Researchers focusing on plant-parasitic nematodes, especially on finding new ways of their control, often need to assess basic parameters such as their motility, viability, and reproduction. Traditionally, these assays involve visually counting juveniles and eggs under a dissecting microscope, making this investigation time-consuming and laborious.

**Results:**

In this study, we established a procedure to efficiently determine the motility of two plant-parasitic nematode species, *Heterodera schachtii* and *Ditylenchus destructor*, using the WMicrotracker ONE platform. Additionally, we demonstrated that hatching of the cyst nematode *H. schachtii* can be evaluated using both the WMicrotracker ONE and by assessing the enzymatic activity of chitinase produced during hatching.

**Conclusions:**

We present fast and straightforward protocols for studying nematode motility and hatching that allow us to draw conclusions about viability and survival. Thus, these methods are useful tools for facilitating fast and efficient evaluation in various fields of research focused on plant-parasitic nematodes.

**Supplementary Information:**

The online version contains supplementary material available at 10.1186/s13007-024-01233-z.

## Background

Plant-parasitic nematodes (PPN) are significant pathogens affecting nearly all major agricultural crops [1]. Given their economic importance, many nematologists have focused on monitoring PPN in the field or studying their biology and plant-parasite interactions to identify new avenues for plant breeders or potential molecular targets for nematicide development. Various substances or biological agents that can interfere with nematode survival or behaviour have also been studied [2].

This research requires the performance of assays to evaluate basic characteristics such as survival, motility, and hatching of PPNs. Traditionally, these assays involve visually observing and counting nematodes and eggs under a dissecting microscope, making them time-consuming and laborious [3]. Several high-throughput methods for performing similar assays have been optimized for the model nematode *Caenorhabditis elegans* [4] and, to some extent, for some species of mammalian parasitic nematodes [5–7]. These methods are based on various principles, including automatic image acquisition and analysis [8, 9], large object flow cytometry systems [10], and many others. While some of these methods require expensive machines, many can be performed using relatively affordable or common laboratory equipment. Surprisingly, attempts to adapt such methods for PPN appear to be relatively rare.

In this work, we present a simple method for evaluating the motility of nematodes using the WMicrotracker ONE. This method provided reliable results for both motile infective juveniles (J2) of the sedentary cyst nematode *Heterodera schachtii* and the migratory endoparasitic nematode *Ditylenchus destructor*, suggesting that the platform is compatible with various PPN species. Furthermore, we describe two fast and robust methods for determining the hatching of *H. schachtii* – one utilizing the WMicrotracker ONE and the other based on measuring the activity of the enzyme chitinase.

## Methods

### Nematode cultivation

#### Maintenance of *Heterodera schachtii*

The stock culture of nematodes was maintained on mustard (*Sinapsis alba* cv. Albatros) roots grown in vitro on modified Knop media according to previously published protocols [11]. Mustard seeds were sterilized by successive treatment with 70% ethanol (1 min), 1.3% NaClO (5 min) and 96% ethanol (1 min) and washed 3 times with sterile double distilled water (ddH_2_O). Seeds were allowed to germinate on 0.8% H_2_O agar for 2 days in the dark at 25 °C, and healthy seedlings (3 per plate) were then transferred onto 15 cm Petri dishes containing modified Knop medium supplemented with 3% sucrose [11]. The plants were subsequently grown at 25 °C under a 16-hour light regime. After 2–3 weeks, each plate was inoculated with approximately 300 *H. schachtii* J2. The infected plates were kept at 25 °C in the dark. Mature cysts of the nematodes could be collected from the infected plates after approximately 2 months, when the worms had completed their life cycle, and females had transformed into mature cysts that were apparent on the roots.

#### Maintenance of *Ditylenchus destructor*

The initial population of *D. destructor* was extracted from a hop plant (Central Bohemia/CZ) and maintained on carrot discs according to previously published protocols [12]. Briefly, to prepare the discs, the carrots were surface-sterilized with 1% NaClO for 30 min, washed in sterile water in the hood, peeled and cut into ca. 1.5 cm pieces with sterilized equipment. The discs were placed on 10 cm Petri dishes and kept at 25 °C in the dark until white callus specks were apparent (approximately 4 weeks). Plates that were not used immediately were stored in the refrigerator. The plates were inoculated with 50–60 nematodes per disc. In our experience, the best time to collect healthy populations for experiments is after 2–4 weeks at 25 °C, when the discs start to develop brown colouration.

#### Collecting nematodes for experiments

The motile *H. schachtii* J2 required for the tests were collected from funnels filled with 3 mM ZnCl_2_ to increase the hatching rate of the nematodes [13]. Approximately 300 cysts were placed in a sieve (60 μm mesh size) in the funnel so that it was approximately half covered with the liquid. The hatched J2 passed through the sieve and settled at the exit of the funnel, which led to a silicone tube closed with a clip. By opening the clip, the hatched J2 could be easily collected. The best time to collect a healthy population of J2 for the experiment was between 3 and 10 days after dissection of the cysts. Juveniles can be collected repeatedly from a funnel.

For experiments with *D. destructor*, 5 ml of sterile ddH_2_O was added to plates containing infected carrot discs. Nematodes naturally migrate from the discs into the water. To increase the yield, the discs were partially submerged in sterile ddH_2_O for approximately 30 min. The liquid containing nematodes was transferred from the plates to Eppendorf tubes. In case any debris from the carrot discs was collected, the nematodes were washed several times with water prior to the experiment (by allowing the nematodes to settle on the bottom of the tubes and exchanging the liquid). Populations containing a mixture of different developmental stages were used for the experiments.

The concentration of nematodes was determined by counting the number of living nematodes in 3 10 µL drops. The suspension was further diluted with sterile ddH_2_O to achieve the desired final concentration.

### Evaluation of nematode motility using WMicroTracker ONE

The WMicrotracker ONE device (Phylumtech S.A.) emits an infrared beam that passes through the wells of a microtiter plate. Moving animals scatter light, and interference is subsequently detected. The instrument evaluates the activity in all wells continuously and then displays the number of these interferences (“activity counts”) per user-defined time interval (“bin”). All the data presented in this study show the number of activity counts detected in wells in 30-minute bins.

**Fig. 1:**
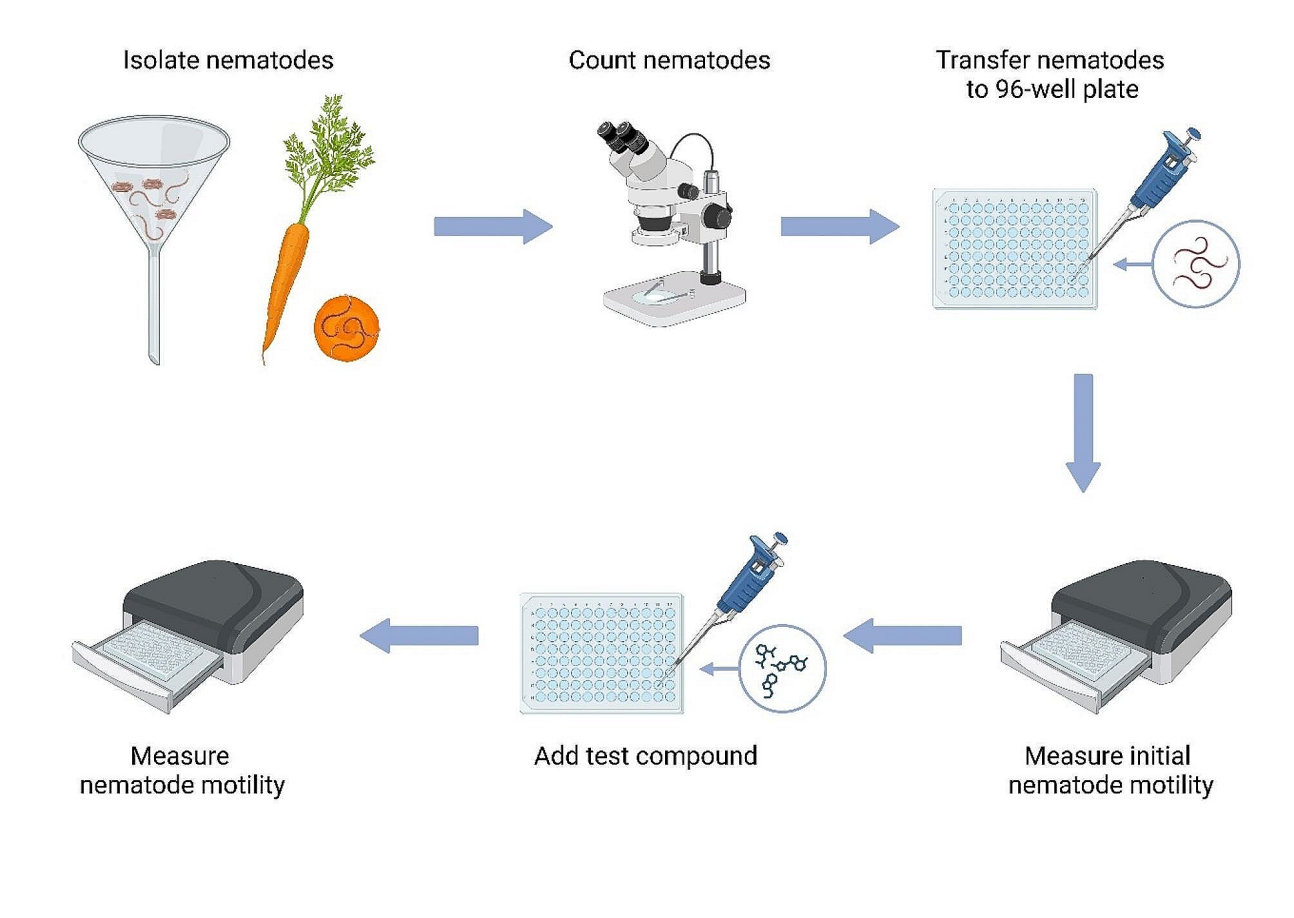
Workflow—evaluation of nematode motility using WMicrotracker ONE [created with BioRender.com]

The workflow of the experiment is schematically displayed in Fig. 1. The suspension was distributed into U-bottom 96-well plates (54 µL per well). The plates were kept in an incubator set to 20 °C for 20–30 min prior to the measurement to allow the nematodes to settle on the bottom of the wells. Afterwards, the plates were placed into WMicrotracker ONE device, and the initial motility of the worms was recorded for 30 min. The device was operated according to the manufacturer’s instructions. Six microliters of the chemicals used as a positive control (i.e., a substance that decreases motility; sodium hypochlorite and sodium azide at a concentration 10 times greater than the desired final concentration selected based on a pilot experiment) or sterile ddH_2_O (negative control) was added to each well (at least 4 wells per condition), and the motility of the populations was remeasured using WMicrotracker ONE at different time points. Between the measurements, the experimental plates were sealed with parafilm or PCR seal, kept at 20 °C, and gently shaken on an orbital shaker (150 rpm) to ensure proper air exchange in the liquid.

### Evaluation of *Heterodera schachtii* hatching

**Fig. 2:**
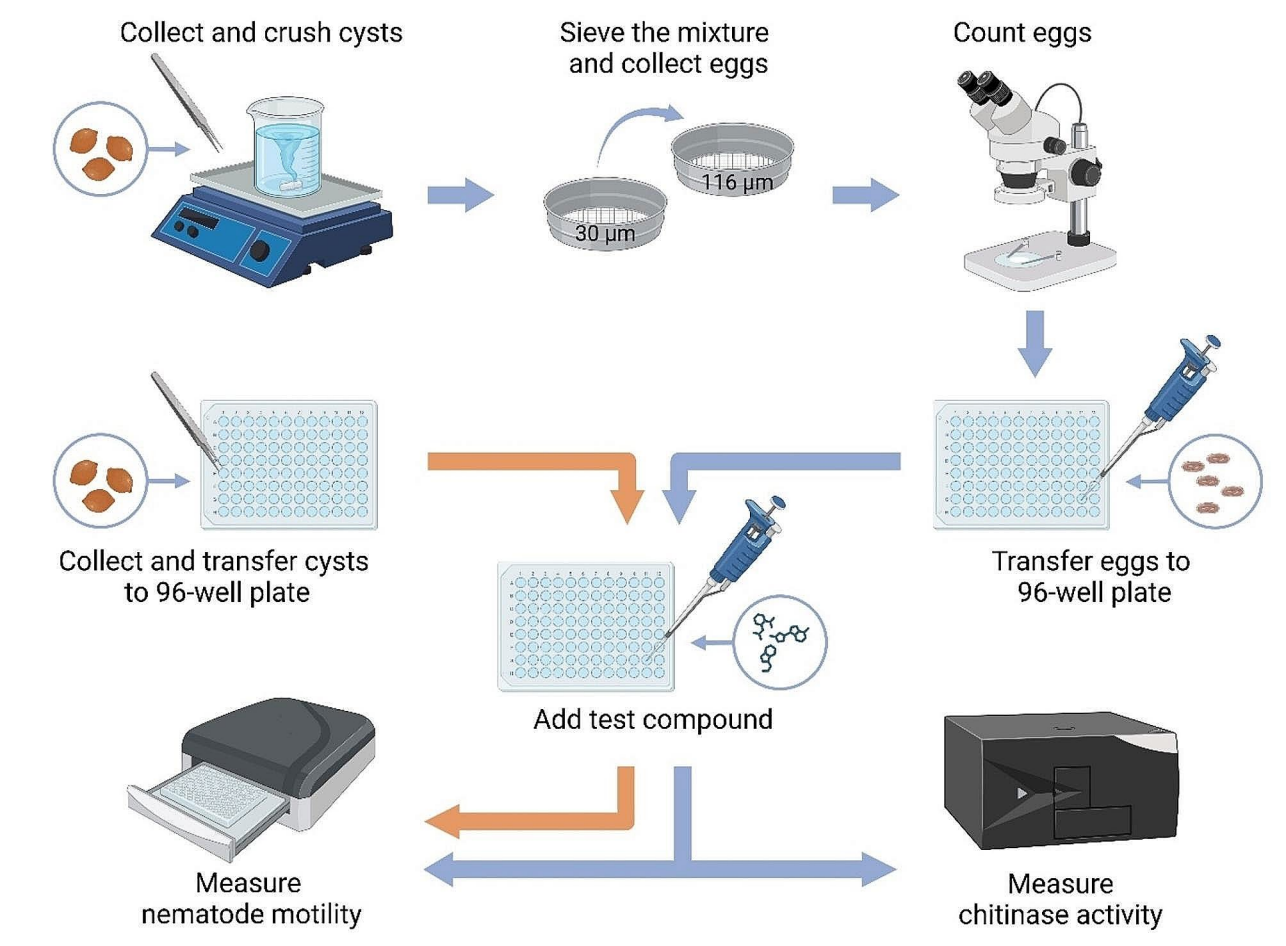
Workflow—evaluation of *Heterodera schachtii* hatching using both WMicrotracker ONE and the chitinase assay [created with BioRender.com]

The workflow of these experiments is schematically displayed in Fig. 2.

#### Measuring the movement of J2 emerging from cysts using WMicrotracker ONE

The wells of a U-bottom 96-well plate were filled with 54 µL of sterile ddH_2_O or 3 mM ZnCl_2_, a known stimulant of nematode hatching [13, 14]. Three cysts were collected from the maintenance plate and placed into each well, while trying to ensure that cysts of similar size and colour were evenly distributed across the wells. Cyst colour is an indicator for maturity. Darker cysts tend to be more mature, and juveniles typically start emerging from them sooner. After measuring the initial motility on WMicrotracker ONE (which should be close to 0 because no juveniles have yet emerged), 6 µL of the chemical used as a positive control (i.e., a substance that decreases hatching; ethanol at a concentration 10 times higher than the desired final concentration selected based on a pilot experiment) or 6 µL of sterile ddH_2_O (negative control) was added to each well. Due to the greater inherent variability of this assay, at least 8 wells per condition were used. The experimental plates were kept under the same conditions and remeasured at different time points, as described in Sect. “Evaluation of Nematode Motility using WMicroTracker ONE”.

#### Measuring hatching using WMicrotracker ONE

Approximately 300 cysts were collected from maintenance plates or retrieved from funnels previously used for other experiments and placed into a 100 ml glass bottle filled with 3–5 ml of sterile ddH_2_O or 3 mM ZnCl_2_. A medium-sized stirring bar was added to the bottle, and the cysts were crushed on a magnetic stirrer (1000 rpm, 5 min). The suspension was passed through a sieve (30 μm pore size) to remove smaller debris and some J2 that had already hatched inside of the cysts. The sieve was placed bottom up on a piece of mesh (116 μm pore size) and washed with 3–5 ml of ddH_2_O. The liquid passing through the mesh was collected. This step removes larger debris. The final suspension was enriched in eggs but was not completely clean, as some J2 and mid-sized debris were also collected. The concentration of eggs was determined by counting the number of intact eggs in three 10 µL drops under a microscope. Approximately 50 eggs per well were used. The experimental plates were prepared, stored, and measured as described in Sect. “Measuring the Movement of J2 Emerging from Cysts using WMicrotracker ONE”.

#### Evaluation of hatching by the chitinase assay

This assay is based on measuring the enzymatic activity of chitinase [14]. The enzyme is produced by hatching juveniles to dissolve the chitin-containing eggshells. Its activity, which correlates to the number of viable juveniles, can be measured by the addition of a fluorogenic substrate.

The egg suspension was prepared as described in Sect. “Measuring Hatching using WMicrotracker ONE”. A total of 400–800 eggs per well were used, and heat-killed eggs (55 °C, 2 h, these conditions were adapted from [15]) served as a positive control. The experimental plates were stored as indicated in the previous sections for 7 days. Afterwards, 20 µM of the chitinase substrate (4-methylumbelliferyl β-D-N, N′,N″-triacetylchitotrioside, stock diluted in DMSO) was added, and the plates were incubated at 37 °C for 1 h. Alkaline buffer (1 M glycine, 1 N NaOH, pH = 10.6) was added at a 1:2 ratio (i.e., 30 µL to 60 µL of liquid in our case). The fluorescence was measured on a TECAN Infinite PRO plate reader (λex 365 nm, λem 460 nm, gain manually set to 65).

### Data analysis

The data were analysed in GraphPad Prism. All the data are displayed as the mean ± standard deviation (SD). Each well containing the same condition on the same plate was considered a technical replicate. Independent experiments performed in the same manner were considered biological replicates. The data were analysed by repeated measures two-way analysis of variance (RM ANOVA) followed by Dunnet’s post hoc test. To assess the quality of the assay, the Z factor [16] was calculated from the mean values and standard deviations using the following formula:

$$Z=1 - \frac{{\left( {3 \times SD\,of\,sample} \right)+\left( {3 \times SD\,of\,control} \right)}}{{mean\,of\,sample - mean\,of\,control}}$$

## Results and discussion

Nematologists routinely need to evaluate basic parameters such as worm motility, viability, and reproduction. These experiments can become laborious and time-consuming, especially when screening large sets of compounds, biological substances etc. Here, we present several fast, robust, and straightforward assays with the potential to be developed into semi- or high-throughput screening tools. The assays described below are based on two different principles described in parts 2.2. and 2.3.3.

### WMicrotracker ONE is a suitable tool for measuring the movement of plant-parasitic nematodes

**Fig. 3:**
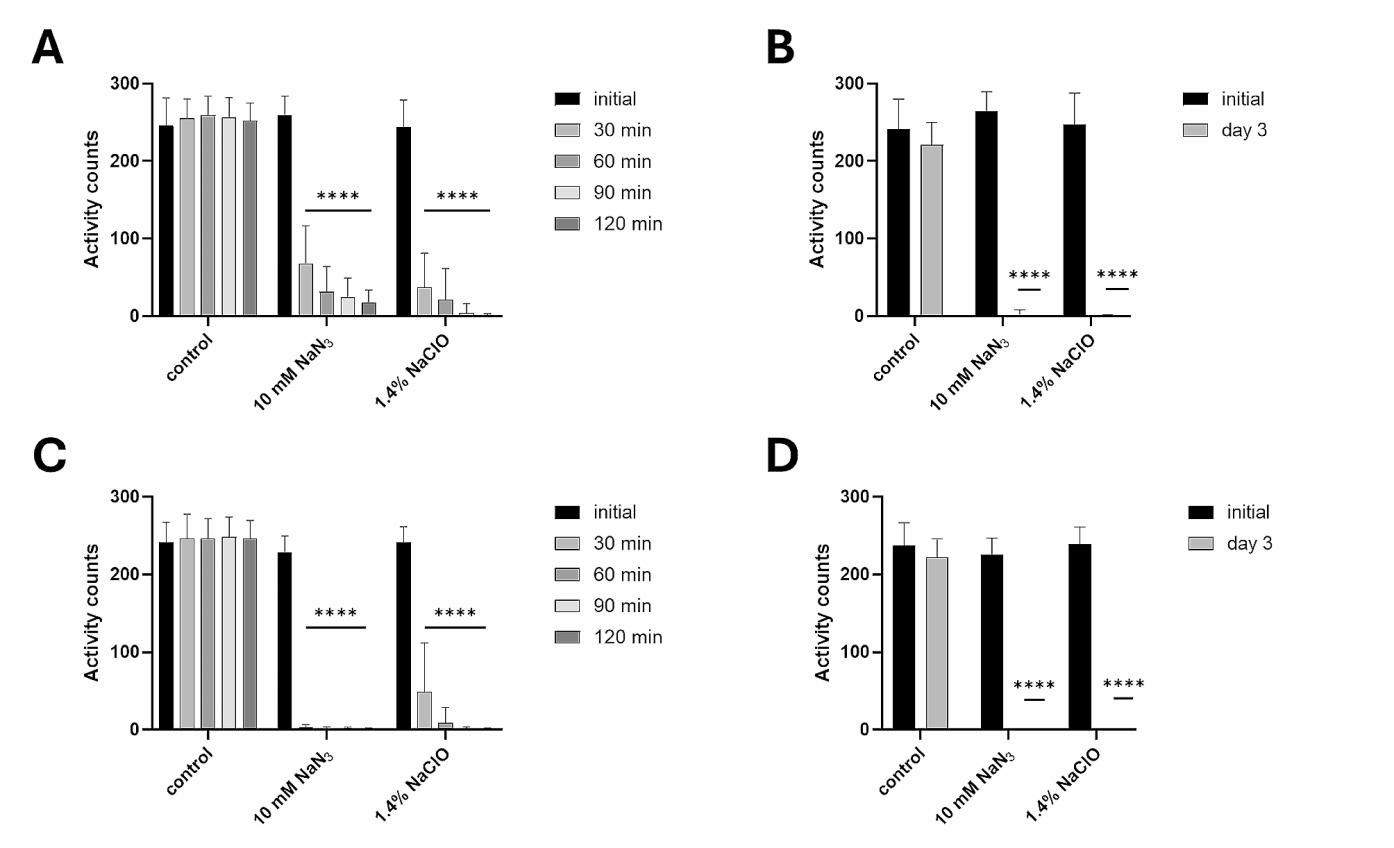
Measuring the motility of plant-parasitic nematodes using the WMicrotracker ONE. (**A**) Effect of short (2-hour) exposure to 2 toxic chemicals (NaN_3_ and NaClO) on the movement of *Ditylenchus destructor* (mixed age). The graph shows the means + SDs from 6 biological (at least 29 technical) replicates. (**B**) The effect of longer exposure (day 3) to 2 toxic chemicals on the movement of *D. destructor* (mixed age). The graph shows the means + SDs from 8 biological (at least 55 technical) replicates. (**C**) The effect of short (2-hour) exposure to 2 toxic chemicals on the movement of *Heterodera schachtii* J2. The graph shows the means + SDs from 7 biological (at least 44 technical) replicates. (**D**) The effect of longer exposure (day 3) to 2 toxic chemicals on the movement of *H. schachtii* J2. The graph shows the means + SDs from 4 biological (at least 29 technical) replicates. In all graphs, asterisks indicate statistically significant differences compared to the negative control at the indicated time points (**** *p* < 0.0001; two-way RM ANOVA, Dunnett´s multiple comparison test)

The WMicrotracker ONE was previously successfully used to analyse the locomotion and behaviour of not only the free-living model nematode *C. elegans* [17] but also multiple species of mammalian parasitic helminths [7, 18], as well as insect and zebrafish larvae [19]. Our experiments (Fig. 3) show that the platform can be used in a similar manner for PPN, including both migratory species and motile developmental stages of sedentary species, which are generally considered the most economically important [1].

To validate the method, we exposed the experimental populations of *D. destructor* (mixed population) and *H. schachtii* (infective J2) to 2 toxic chemicals, sodium azide (10 mM) and sodium hypochlorite (1.4%). We chose to use these compounds as a positive control due to their reliable effect. During the selection process, we also tested the effect of a variety of commonly used anthelmintic drugs, such as levamisole, ivermectin and mebendazole, with unsatisfactory results (data not shown). These drugs are likely unable to efficiently penetrate the cuticle of nematodes [20]. Better results would presumably be obtained if the nematodes were actively feeding and ingesting the substance during the exposure [21]. It is important to consider this when, for example, testing new promising substances for possible nematicidal activity.

We measured the motility of the treated and non-treated nematodes for 2 h immediately after the addition of the compounds and again 3 days after treatment (Fig. 1). As expected, both species showed a rapid decline in movement after exposure to both substances. After 30 min, the activity was reduced by 73.8% (from mean 258.9 activity counts to 67.7) in populations of *D. destructor* treated with NaN_3_, 84.9% (from mean 243.9 activity counts to 36.9) in *D. destructor* treated with NaClO, 98.9% (from mean 227.8 activity counts to only 2.4) in *H. schachtii* exposed to NaN_3_, and 79.7% (from mean 241.6 activity counts to 49) in *H. schachtii* in NaClO. Less than 1 mean activity count could be detected in populations of both nematode species treated with both substances after 3 days. At the same time, nematode motility in the negative controls remained consistent, with no decrease in activity counts detected in the short-term experiment and only a small decrease in motility (6–8% on average) after 3 days.

According to our results, approximately 100–150 worms per well should be used for smaller and less active nematodes such as *H. schachtii* J2. For *D. destructor* and other more active PPN species, 30–50 nematodes per well are sufficient. Using plates with round bottoms allows nematodes to accumulate more closely and further stimulate each other’s movement by touch, resulting in higher detected activity counts than in plates with flat bottoms. This finding is in agreement with the information published on the website of Phylumtech S.A., the manufacturer of WMicrotracker ONE. The wells should contain approximately 40–100 µL of liquid in total. The bottom of the plate needs to be fully covered, but too large volumes of liquid in wells could hinder aeration of the suspension, especially during longer experiments.

The assay is very efficient and easy to evaluate, especially in comparison to the visual counting of moving nematodes under a microscope. Nematodes cultivated in vitro can be harvested in relatively large quantities. This makes the assay suitable for high-throughput screening. To assess the quality of the assay, we calculated the Z factor [16]. Values exceeding 0.5 (a threshold for the assay to be considered excellent) were achieved both overall and in all individual replicates, in longer experiments as well as in shorter assays, at 90 min and longer for *H. schachtii* and 120 min for *D. destructor* (supplementary Table 1). This indicates that the assay is reliable and could presumably be used for screening larger libraries of compounds or other substances for nematicidal activity, similar to what was shown for nematodes parasitizing mammals [7].

### Both the WMicrotracker ONE and the chitinase assay are suitable for evaluating the hatching of *Heterodera schachtii*

The hatching rate of a PPN and the ability of a (putative) agent to decrease it are important parameters. *H. schachtii* cysts can contain hundreds of eggs that remain vital in the soil for many years and are awaiting optimal conditions to hatch and infect a host plant [22]. Thus, we established two streamlined protocols allowing us to reliably evaluate reproductive capacity of PPN.

To obtain the best possible results, an important step in preparing these assays is acquiring a clean egg suspension. Unwanted debris can hinder the experiment in several ways. Poor visibility of the eggs in the suspension can make accurate determination of their concentration difficult. Larger pieces of debris tend to block the pipette, resulting in uneven distribution of the egg suspension. Reducing the amount of debris is especially crucial when using the WMicrotracker ONE device, where extra material present in wells could interfere with proper signal detection.

The isolation of clean eggs from *C. elegans* can be performed simply by dissolving juveniles and adult nematodes in bleach [23]. However, this process is not suitable for PPN, where eggs are usually extracted by passing the suspension through a series of sieves or using floating and centrifugation [3, 24]. In the case of *H. schachtii*, we mechanically crushed the cysts and then carefully passed the suspension through 2 sieves with different pore sizes (see Sect. “Measuring Hatching using WMicrotracker ONE” for details). While the final suspension was not completely debris or juvenile free, it was sufficient for us to observe reproducible trends among biological replicates and statistically significant differences between positive and negative controls using both methods described.

#### The chitinase assay

The chitinase assay was originally described for evaluating hatching in *C. elegans* [25], and a modified version of this assay was used to test the toxic effects of various compounds on *C. elegans* [26, 27]. As mentioned above, the principle of the assay involves the addition of the fluorogenic substrate 4-methylumbelliferyl β-D-N, N′,N″-triacetylchitotrioside. The reagent is cleaved by the nematode-produced enzyme chitinase, leading to the release of fluorescent 4-methylumbelliferone that can be detected.

**Fig. 4:**
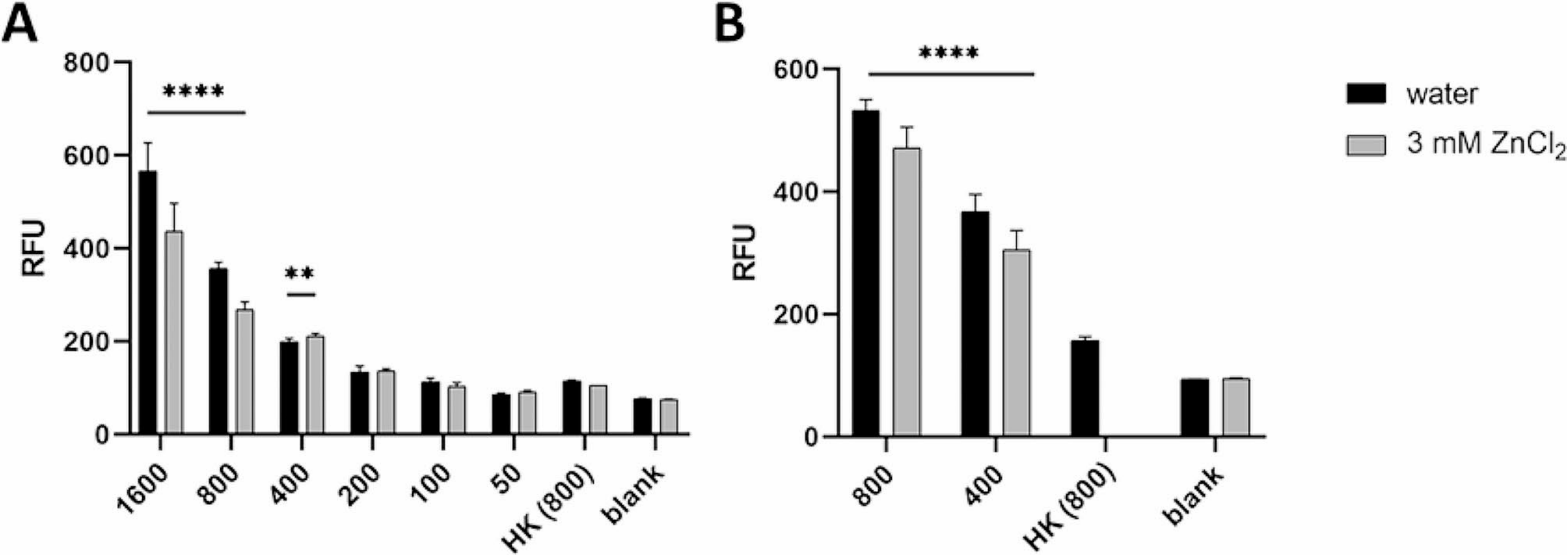
Evaluation of egg hatching of *Heterodera schachtii* using the chitinase assay. Panels **A** and **B** depict 2 representative experiments with different egg population sizes (X-axis; 1600–50 range) and heat-killed eggs (HK; 55 °C for 2 h) as a positive control. The data are displayed as the means + SDs of 2–4 technical replicates. Refer to supplementary information S1 and S2 for results from repeated experiments. In all graphs, asterisks indicate statistically significant differences compared to nonviable, heat-killed eggs (**** *p* < 0.0001; ** 0.05 > *p* > 0.001; two-way ANOVA, Dunnett´s multiple comparison test). RFU – relative fluorescent units

Our results show that the assay can also be used with PPN eggs (Figs. 4, S1 and S2).

As a positive control, we used heat-inactivated eggs We avoided using chemicals due to possible unwanted interactions. For instance, sodium azide, which was used as a positive control in previous assays, was reported to directly inhibit the activity of some chitinases [28]. On the other hand, sodium hypochlorite, our other substance used as a positive control, could negatively interact with ZnCl_2_, in which the eggs were incubated.

Our rationale for using both water and ZnCl_2_ was based on the reported ability of zinc salts to stimulate the hatching of several members of the genus *Heterodera*, including *H. schachtii* [13, 14]. Eggs of some species of PPN, including *Heterodera* spp., usually only start to hatch after they detect suitable environmental conditions and/or cues from nearby hosts. In our experiment, we observed a noticeable increase in the number of juveniles after 1 week of incubation of the eggs in both water and ZnCl_2_ (Fig. S3), suggesting that hatching occurs even without additional stimuli. An increased number of juveniles correlated with increased detected activity of the enzyme chitinase.

In the initial experiments (Figs. 2A and S1), we determined that a concentration of 400 eggs per well was sufficient to achieve a reproducible, statistically significant difference between the positive and negative controls. The signal detected was approximately 1.7–2 times greater (increase of 71–125 RFU – relative fluorescence units) for the eggs incubated in both water and ZnCl_2_ than for the heat-killed positive control. The difference was more obvious when more eggs were used, with 1600 eggs per well providing a signal more than 4 times greater than that of the positive control. Nevertheless, as the process of egg preparation is relatively laborious, using as few eggs as possible is desirable. To ensure that 400 eggs were indeed sufficient, we repeated the experiment 5 more times with 800 and 400 eggs only (Figs. 2B and S2). In all experiments, we again observed a statistically significant increase in signal intensity in wells containing 400 eggs compared to the positive control (between 161 and 450 RFU increase in water and 124 and 245 RFU increase in ZnCl_2_-incubated eggs). Again, the difference was more pronounced when 800 eggs were used (between 351 and 835 RFU increase in water and 246 and 544 RFU increase in ZnCl_2_-incubated eggs).

Although the variance among the technical replicates within one plate was relatively low, we observed notable differences in the detected signal strength among the biological replicates. This can likely be attributed to factors such as differences in the age of the maintenance plates from which *H. schachtii* cysts were collected, inaccuracies in determining the egg concentration resulting in slight inconsistencies in egg population size, and other similar sources of biological variability. Therefore, we recommend presenting the data from each repetition as an individual graph. All the biological replicates followed the same significant trend, validating the reliability of the assay.

Interestingly, we observed that the detected chitinase activity was slightly lower in the wells with ZnCl_2_ than in the wells where the eggs were incubated in water. A possible explanation could be the direct interference of zinc with the enzyme. Heavy metals, including zinc, were previously reported to inhibit the activity of some chitinases [29]. Nevertheless, this effect is clearly not significant enough to affect the ability of the juveniles to hatch, and the differences in chitinase activity in ZnCl_2_-incubated eggs and positive controls are reproducibly statistically significant. Therefore, researchers may use both depending on their specific needs.

Although this assay could be easily adapted for many other nematode species, an obvious drawback could be its incompatibility with those species that can produce chitinase in life stages other than during hatching. For these cases, further optimization of the egg cleaning process would likely be necessary to ensure that the suspension used for the experiment contained only eggs. For example, depending on egg size and other species-specific characteristics, in some PPN it could be possible to obtain better separation of eggs using carefully selected sieve sizes. Using one of the many egg floatation protocols available [30, 31] or their combination with sieves can further improve the results. For some nematodes, it might also be possible to adapt a protocol similar to bleaching in *Caenorhabditis elegans* [23], and further chemically purify a roughly pre-cleaned suspension. Moreover, the use of various lures [32] or microfluidic devices [33] might be a good option in some cases. Many laboratories already have well-optimized protocols for the isolation of eggs of the species on which they are focused. Another limiting factor is the need for a relatively large number of eggs. While the assay is undoubtedly more efficient than visual counting of hatched juveniles, one experiment typically requires several thousand eggs. This might make collecting a sufficient number of cysts for a large experiment quite tedious and unsuitable for high-throughput experiments. The assay might also not be useful in cases where only a limited amount of material is available, such as for monitoring the reproductive capacity of *Heterodera* spp. collected from the field. This is why we decided to evaluate the hatching of *H. schachtii* by detecting an increase in motility caused by the presence of newly hatched active juveniles via the WMicrotracker ONE.

#### Measuring the motility and hatching of *Heterodera schachtii* using the WMicrotracker ONE

**Fig. 5:**
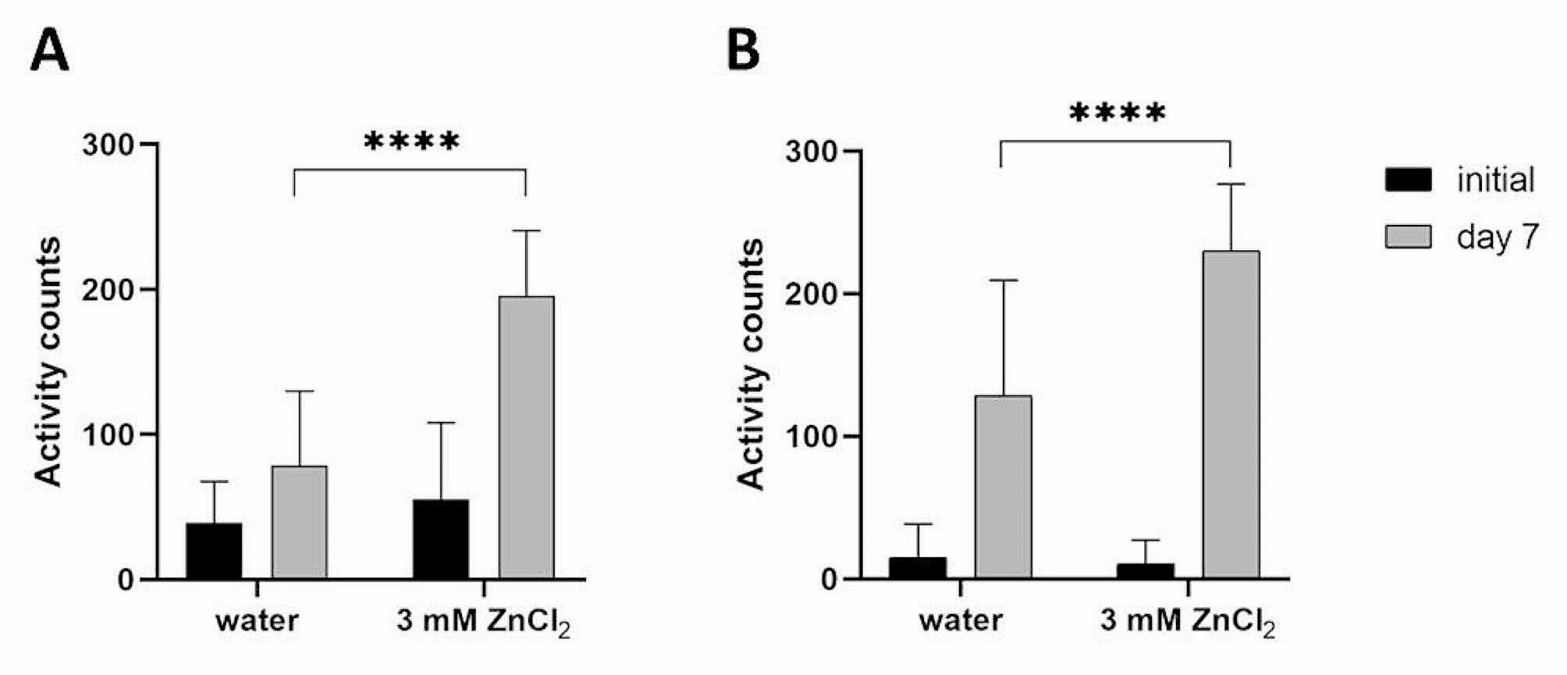
*Heterodera schachtii* eggs and cysts incubated in ZnCl_2_ for 7 days showed significantly greater activity than those incubated in water (measured by WMicrotracker ONE). **A** Results from isolated eggs, 50 per well. The data are shown as the means + SDs from 5 biological (at least 16 technical) replicates. **B** Results from intact cysts, 3 per well. The data are shown as the means + SDs from 3 biological (28 technical) replicates. In both graphs, asterisks indicate statistically significant differences compared to the negative control (**** *p* < 0.0001; two-way RM ANOVA, Dunnett multiple comparison test)

We tested two different setups—measuring the increase in motility in wells with intact cysts and in wells containing the egg mixtures prepared in the same way as for the chitinase assay. In both cases, we again compared the data for populations incubated in water and in ZnCl_2_. Here, the reported stimulatory effect of zinc salt on the hatching of *Heterodera* spp. was clearly apparent (Fig. 5). In wells containing eggs incubated in ZnCl_2,_ we detected a 60% increase in motility compared to water (195.9 mean activity counts compared to 78.8). For intact cysts, the difference was 44% (229.9 mean activity counts compared to 128.4). Therefore, we used ZnCl_2_ in all subsequent experiments.

**Fig. 6:**
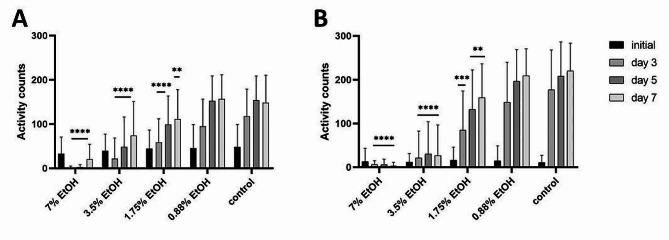
Effect of different concentrations of ethanol (7% − 0.88%) on *Heterodera schachtii* activity measured with WMicrotracker ONE. **A** Results from isolated eggs, 50 per well, incubated in ZnCl_2_. The data are shown as the means + SDs from 8 biological (64 technical) replicates. **B** Results from intact cysts, 3 per well, incubated in ZnCl_2_. The data are shown as the means + SDs from 4 biological (32 technical) replicates. In both graphs, asterisks indicate statistically significant differences compared to the water-treated negative control at the indicated time points (**** *p* < 0.0001; *** 0.001 > *p* > 0.0001; ** 0.05 > *p* > 0.001; two-way RM ANOVA, Dunnett´s multiple comparison test)

To validate the robustness of the two assay setups, various concentrations of ethanol were used as a positive control, demonstrating the dose-dependent effect of the substance at various time points (Fig. 6). For both setups, we observed greater variability among the technical replicates, while the effect remained consistent among the biological replicates. We assume that this is due to inherent variability among the cysts and eggs and recommend using at least 8 technical replicates per condition to mitigate this issue. Furthermore, we noticed that the age of the plates from which the cysts were collected can significantly influence how soon hatching starts. Therefore, we recommend using maintenance plates of approximately the same age across all biological replicates.

In the setup with intact cysts, we recommend using 3 cysts per well. During pilot experiments (not shown), we established that using fewer than 3 cysts further increases the variability among technical replicates. More than 3 cysts in the well seem to interfere with the instrument´s ability to measure the activity properly, resulting in high variability and unexpected lower activity counts in some wells, even though visual examination in a microscope revealed the presence of motile J2.” For the egg suspension, we recommend using 50 eggs per well. The suspension obtained from crushed cysts, while enriched in eggs, also contained some J2 and mid-size debris. The presence of J2 at the beginning of the test results in some activity being detected in the wells during the initial measurement prior to adding the substances. These J2 tend to become inactive relatively quickly, likely due to stress caused during the preparation process. Accurately assessing the actual increase in the activity over time as the J2 hatch, while nematodes that were already present became inactive, might become difficult when a more concentrated mixture (with a stronger initial signal) is used. Another factor that might hinder the acquisition of reliable data is the presence of debris, which might interfere with beams passing through the wells. Using 50 eggs per well limits the number of unwanted juveniles and debris sufficiently, allowing the detection of a clear increase in activity counts over time due to hatching events.

The difference between the positive and negative controls was reproducible and statistically significant in all biological replicates. For intact cysts, we observed a gradual increase in activity counts from approximately 10–16 during the initial measurement in all conditions to 220.1 mean activity counts in wells containing negative control after 1 week. For ethanol-treated cysts, the values obtained at the same time point were 209.2, 159.2, 26.7 and 2.9 for 0.88, 1.75, 3.5 and 7% ethanol, respectively. The effect was dose-dependent and ranged from none or marginal to severe. For isolated eggs, the initial mean activity counts ranged from approximately 33 to 50 under all conditions for the reasons outlined above. The movement rates in the wells gradually increased to approximately 150 mean activity counts at days 5 and 7 in both the negative control wells and the wells treated with 0.88% ethanol. A less prominent increase, to 111.6 and 73.8 mean activity counts after 1 week, was apparent in populations treated with 1.75 and 3.5% ethanol, respectively. The addition of 7% ethanol led to a decrease in the mean activity counts after 1 week compared to the initial value, to only 20.1.

The chitinase assay has two obvious advantages over determining hatching activity via the WMicrotracker ONE. First, the presence of either J2 or debris does not interfere with the readout. Second, the population size of 50 eggs is relatively small and more prone to random error, contributing to greater deviation among technical replicates here than in the chitinase assay. Further cleaning of the suspension might be helpful, although we opted to not focus on this step further, as the assay provided reproducible and statistically significant data. Moreover, unlike in the chitinase assay, the WMicrotracker ONE experiment performed significantly better when ZnCl_2_ was used. Researchers should be mindful of possible unwanted interference of the solution with substances or biological agents they plan to test on cyst nematodes and assess their possible interactions beforehand. This problem will not apply to many other species of PPN that do not require zinc salts to stimulate hatching. We believe that both the chitinase test and the WMicrotracker ONE measurements are compatible with other nematode species. The egg isolation process will need to be modified to account for differences in egg size and other species-specific parameters. Many laboratories already have well-optimized protocols for the isolation of eggs of the species on which they are focused.

Overall, the established protocols are valuable, as both the chitinase assay and the WMicrotracker ONE assay are significantly less labor intensive than commonly used procedures and allow the testing of dozens of substances/conditions. The protocols are a good basis for further optimizations and adjustments that might even make them suitable for high-throughput screening. For example, while we worked with a 96-well plate format only, WMicrotracker instruments, depending on the model, also allow switching to a 384-well plate format. The same is true for the plate readers used for the chitinase assay. The advantages of the presented methods over other very promising modern approaches, such as automated image acquisition and image analysis [34], include the ease of data analysis and the significantly lower cost of the instrumentation needed. As outlined above, the current bottlenecks are the relative laboriousness of preparing the egg suspensions and/or collecting the cysts, the biological variability, and, in the case of setups that work better with ZnCl_2_, the necessity to perform prior tests.

## Conclusions

In this study, we optimized several assays to evaluate the motility and hatching of PPN. We primarily focused on *H. schachtii* and included the migratory species *D. destructor* for comparison when determining motility. From our experiments, we conclude that the developed methods can also be used with other economically important PPN, either in the presented form or with minor species-specific modifications. The methods are based on the detection of nematode movement in multiwell plates and the quantification of enzyme activity using a fluorogenic substrate. Inspired by protocols previously optimized for the model *C. elegans* and other nematodes, they also proved to be compatible with PPN species. These methods offer a more efficient alternative to commonly used approaches. They can find use in various areas of basic and applied research as well as in laboratories focused on PPN monitoring.

### Electronic supplementary material

Below is the link to the electronic supplementary material.

Supplementary Material 1

## Data Availability

No datasets were generated or analysed during the current study.

## Abbreviations

PPN: Plant-parasitic nematode
ddH_2_O: Demineralized water
J2: Second-stage juveniles
RFU: Relative fluorescence units
