# Supplementary material for: Advanced screening methods for assessing motility and hatching in plant-parasitic nematodes
Source: Plant Methods. 2024 Jul 20;20:108. doi: 10.1186/s13007-024-01233-z (PMC11264999; doi:10.1186/s13007-024-01233-z)
Supplement: Supplementary file 1 — Supplementary Material 1 [file 13007_2024_1233_MOESM1_ESM.docx]

**Supplementary Information**

**TITLE: Advanced Screening Methods for Assessing Motility and Hatching in Plant-Parasitic Nematodes**


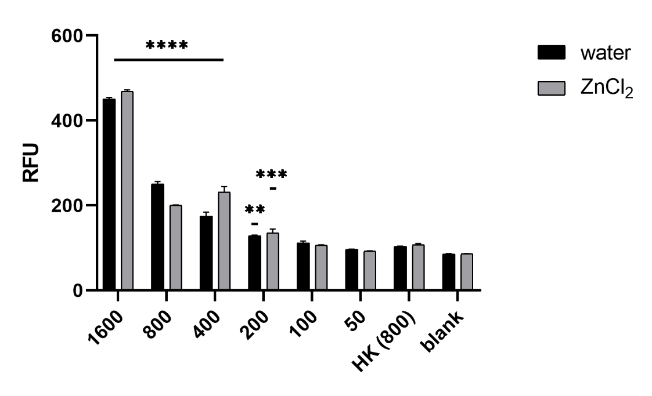


Supplementary figure S1: Evaluation of egg hatching of *Heterodera schachtii* using the chitinase assay – results from repeated experiment. X-axis: different egg population (1600 – 50 range) sizes and heat-killed eggs (HK; 55 °C 2 hours) as a positive control. Data are displayed as mean + SD of 2 – 3 technical replicates. Asterisks indicate statistically significant result when compared to non-viable, heat-killed eggs (**** p < 0.0001; *** 0.001 > p > 0.0001; ** 0.05 > p > 0.001; Two-way ANOVA, Dunnett multiple comparison test). RFU – Relative Fluorescent Units.


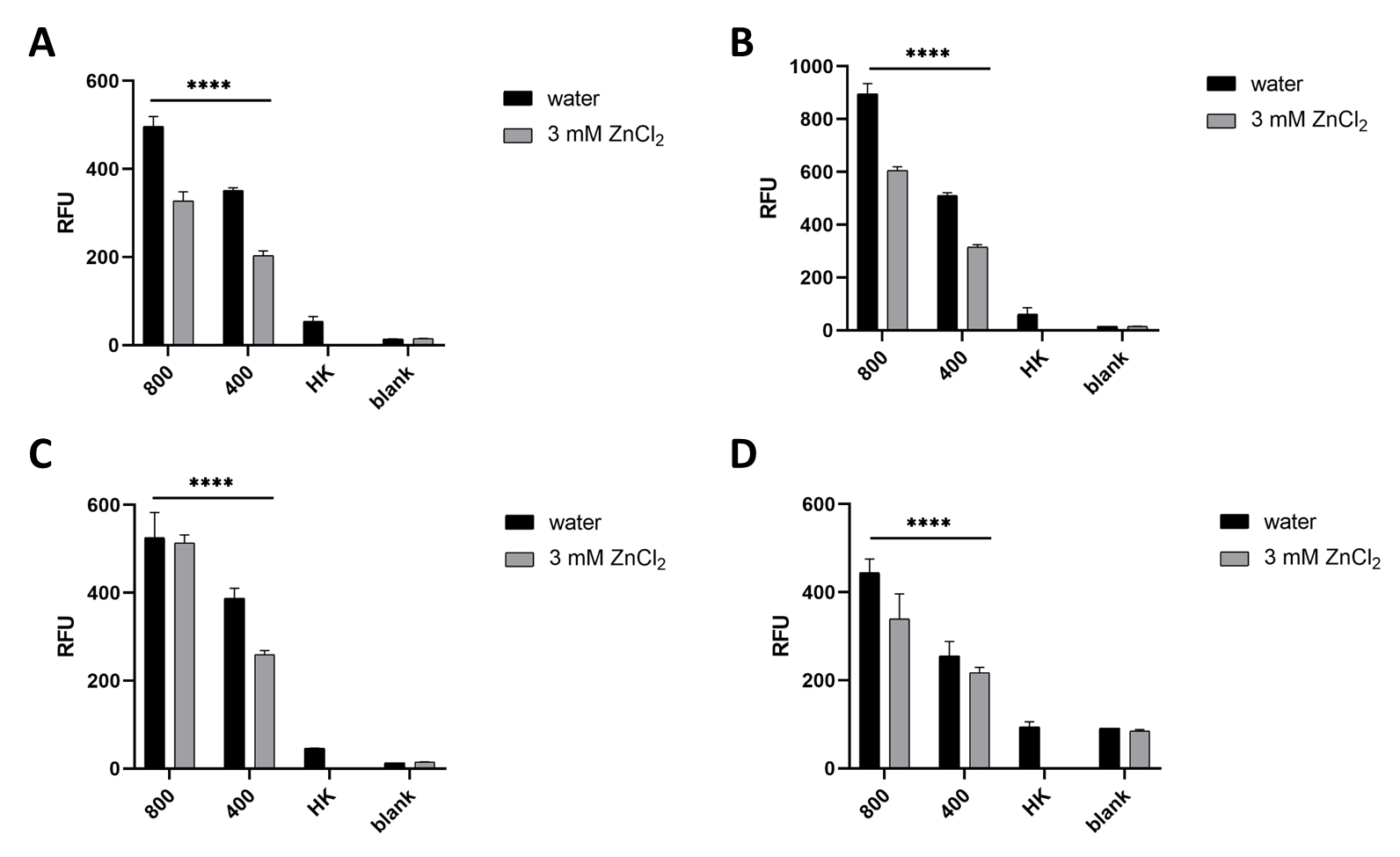


Supplementary figure S2: Evaluation of egg hatching of *Heterodera schachtii* using the chitinase assay – results from 4 repeated experiments (A – D). X-axis: different egg population (400 and 800) sizes and heat-killed eggs (HK; 55 °C 2 hours, 800 eggs) as a positive control. Data are displayed as mean + SD of 2 – 5 technical replicates. In all graphs, asterisks indicate statistically significant result when compared to non-viable, heat-killed eggs (**** p < 0.0001; Two-way ANOVA, Dunnett multiple comparison test). RFU – Relative Fluorescent Units.

Supplementary table 1: Z factor values calculated for each indiviual bological replicate and overall value calculated from data from all experiments pooled together.

| **species** | **compound** | **time point/replicate** | **1** | **2** | **3** | **4** | **5** | **6** | **7** | **8** | **overall** |
| --- | --- | --- | --- | --- | --- | --- | --- | --- | --- | --- | --- |
| *Ditylechus destructor* | NaN_3_, 10 mM | 30 min | 0,4 | 0,3 | 0,3 | -0,2 | -0,1 | 0,5 | -0,3 | 0,0 | -0,2 |
|  |  | 60 min | 0,6 | 0,6 | 0,7 | -0,3 | 0,2 | 0,8 | -0,1 | 0,7 | 0,2 |
|  |  | 90 min | 0,6 | 0,6 | 0,4 | 0,1 | 0,4 | 0,7 | 0,1 | 0,6 | 0,3 |
|  |  | 120 min | 0,6 | 0,6 | 0,7 | 0,7 | 0,6 | 0,8 | 0,6 | 0,8 | 0,5 |
|  |  | 3 days | 0,6 | 0,7 | 0,7 | 0,6 | 0,5 | 0,6 | - | - | 0,5 |
|  | NaClO 1.4% | 30 min | 0,3 | 0,5 | 0,7 | 0,6 | 0,3 | 0,6 | 0,2 | -0,5 | 0,1 |
|  |  | 60 min | 0,6 | 0,7 | 0,8 | 0,4 | 0,7 | 0,8 | -0,1 | 0,1 | 0,2 |
|  |  | 90 min | 0,7 | 0,8 | 0,8 | 0,5 | 0,6 | 0,8 | 0,6 | 0,7 | 0,6 |
|  |  | 120 min | 0,6 | 0,8 | 0,8 | 0,9 | 0,8 | 0,9 | 0,8 | 0,8 | 0,7 |
|  |  | 3 days | 0,6 | 0,7 | 0,7 | 0,6 | 0,7 | 0,6 | - | - | 0,6 |
| *Heterodera schachtii* | NaN_3_, 10 mM | 30 min | 0,5 | 0,8 | 0,9 | 0,8 | 0,5 | 0,7 | 0,8 | - | 0,5 |
|  |  | 60 min | 0,6 | 0,8 | 0,8 | 0,8 | 0,6 | 0,9 | 0,9 | - | 0,6 |
|  |  | 90 min | 0,6 | 0,9 | 0,8 | 0,8 | 0,7 | 0,8 | 0,8 | - | 0,6 |
|  |  | 120 min | 0,7 | 0,8 | 0,9 | 0,9 | 0,6 | 0,8 | 0,9 | - | 0,7 |
|  |  | 3 days | 0,8 | 0,6 | 0,7 | 0,7 | - | - | - | - | 0,7 |
|  | NaClO 1.4% | 30 min | -0,5 | 0,7 | 0,9 | 0,8 | 0,0 | -0,6 | -0,6 | - | -0,5 |
|  |  | 60 min | 0,4 | 0,8 | 0,8 | 0,8 | 0,6 | 0,4 | 0,7 | - | 0,4 |
|  |  | 90 min | 0,6 | 0,9 | 0,8 | 0,9 | 0,7 | 0,8 | 0,9 | - | 0,6 |
|  |  | 120 min | 0,7 | 0,9 | 0,9 | 0,9 | 0,6 | 0,8 | 0,9 | - | 0,7 |
|  |  | 3 days | 0,8 | 0,6 | 0,7 | 0,7 | - | - | - | - | 0,7 |
